# Endoscopic plantar fascia release via dual medial deep fascia approach for refractory plantar fasciitis: an effective, safe, and rapid surgical approach

**DOI:** 10.1007/s00264-025-06499-z

**Published:** 2025-03-17

**Authors:** Jianing Yu, Jinxi An, Sen Wang, Wei Liu, Ziheng Bu, Junchao Huang, Peng Wang, Tao Zhu, Peng Wu, Min Zhu

**Affiliations:** 1 The First Affiliated Hospital of Anhui University of Science and Technology (Huainan First People’s Hospital), Huainan, 232001 Anhui China; 2 Department of Orthopedics, Shanghai Tenth People’s Hospital, School of Medicine, Tongji University, Shanghai, 200072 China; 3 School of Medicine, Anhui University of Science and Technology, Huainan, 232001 Anhui China

**Keywords:** Refractory plantar fasciitis, Endoscope, Arch, Endoscopic approach

## Abstract

**Objective:**

This study aims to evaluate the clinical efficacy of endoscopic plantar fascia release through the modified dual medial deep fascia approach for the treatment of refractory plantar fasciitis.

**Methods:**

A retrospective study was conducted involving 34 patients with refractory plantar fasciitis treated by endoscopic plantar fascia release through the modified dual medial deep fascia approach. Among them, 25 patients had concurrent calcaneal spurs. All patients were followed for a minimum of 12 months. Functional outcomes were assessed using the Visual Analogue Scale (VAS) and the American Orthopaedic Foot and Ankle Society (AOFAS) score, while structural evaluations included the Medial Longitudinal Arch Angle (MLAA), navicular tuberosity height-to-foot length ratio (NH/FL), and the Arch Index (AI). Differences between patients with and without calcaneal spurs were also analyzed.

**Results:**

All patients completed at least 12 months of follow-up, with primary wound healing in all cases. Two patients experienced transient plantar skin numbness and small toe abduction difficulty, which resolved within three months. The VAS score decreased significantly from 6.53 ± 1.19 preoperatively to 1.18 ± 0.76 postoperatively, and the AOFAS score improved from 52.41 ± 5.23 to 93.29 ± 3.91 (both *P* < 0.05), indicating statistical significance. However, changes in the MLAA, NH/FL and AI were not statistically significant. Apart from age differences (49.04 ± 4.41 vs. 34.56 ± 3.13), no significant differences in other scores were observed between the calcaneal spur group and the non-calcaneal spur group at the final follow-up. Moreover, compared to the methods reported in other studies, our study demonstrated a shorter operative time and superior pain and functional outcomes.

**Conclusion:**

The dual medial deep fascia approach for endoscopic plantar fascia release is a safe, quick, effective, and minimally invasive technique that yields favourable clinical outcomes. It has certain advantages compared to other techniques. The presence of calcaneal spurs does not impact postoperative outcomes.

## Introduction

Plantar fasciitis is one of the leading cause of heel pain, accounting for approximately 10% of cases [1]. Despite its prevalence, it has received limited attention. Research indicates that approximately 10% of patients develop refractory plantar fasciitis, defined as cases that remain unresponsive to rigorous conservative treatments, including biomechanical therapy (customized arch supports), specific stretching exercises and night splints, extracorporeal shockwave therapy, corticosteroid injections, and platelet-rich plasma (PRP) injections, after a six-month period [2, 3].This condition significantly impairs patients’ quality of life and frequently necessitates surgical intervention. Surgical intervention is an effective means to treat refractory plantar fasciitis, including open surgery, percutaneous micro-incision, and endoscopic surgery [4, 5]. Among these, endoscopic surgery has become the preferred approach due to its minimally invasive advantage, faster recovery, and better aesthetic outcomes [6, 7]. Surgical approaches can be categorized based on the targeted layer of the fascia: deep fascia approach or superficial fascia approach [8]. The deep fascia approach offers better visualization and facilitates the efficient removal of calcaneal spurs [9]. Traditionally, this method employs both medial and lateral incisions. Previous studies have demonstrated that the dual medial-lateral deep fascial approach is an effective technique for treating refractory plantar fasciitis, consistently achieving favorable clinical outcomes. However, it also has some drawbacks, including procedural complexity, prolonged surgical duration, and high technical demands, and the potential for increased nerve injury risk with the lateral approach [8, 10–12].

In clinical practice, we have found that the dual medial approach may have advantages over the medial and lateral approaches. It allows for easier removal of medial bone spurs and, due to the positional advantages during surgery, may reduce operative time, thereby minimizing patient injury and pain, accelerating recovery, reducing the risk of nerve injury and providing an overall better patient experience [13]. Previously, no one has systematically studied the clinical outcomes of endoscopic plantar fascia release using the dual medial deep fascia approach for treating refractory plantar fasciitis. This study aimed to comprehensively evaluate the clinical outcomes of the dual medial deep fascia endoscopic approach for refractory plantar fasciitis from both functional and structural perspectives. Additionally, given the unclear relationship between calcaneal bone spurs in plantar fasciitis patients and the severity of the condition [14–17], we also analyzed the impact of bone spurs on surgical outcomes.

We hypothesized that this surgical technique is effective, easy to perform, requires shorter operative time, and allows patients to achieve excellent functional recovery while maintaining structural stability.

## Materials and methods

### Subjects

To investigate the surgical efficacy of the dual medial deep fascia approach, we conducted a retrospective study on 34 patients with refractory plantar fasciitis who underwent this surgical procedure. We combined functional and structural scores to assess the clinical outcomes of endoscopic plantar fascia release via the dual medial deep fascia approach.

All surgical procedures were performed by the same surgeon following informed consent from all patients, which included detailed explanations regarding potential surgical risks. This study was approved by the Institutional Ethical Committee of local hospital (ID 24K79).

#### Inclusion criteria

**Fig. 1:**
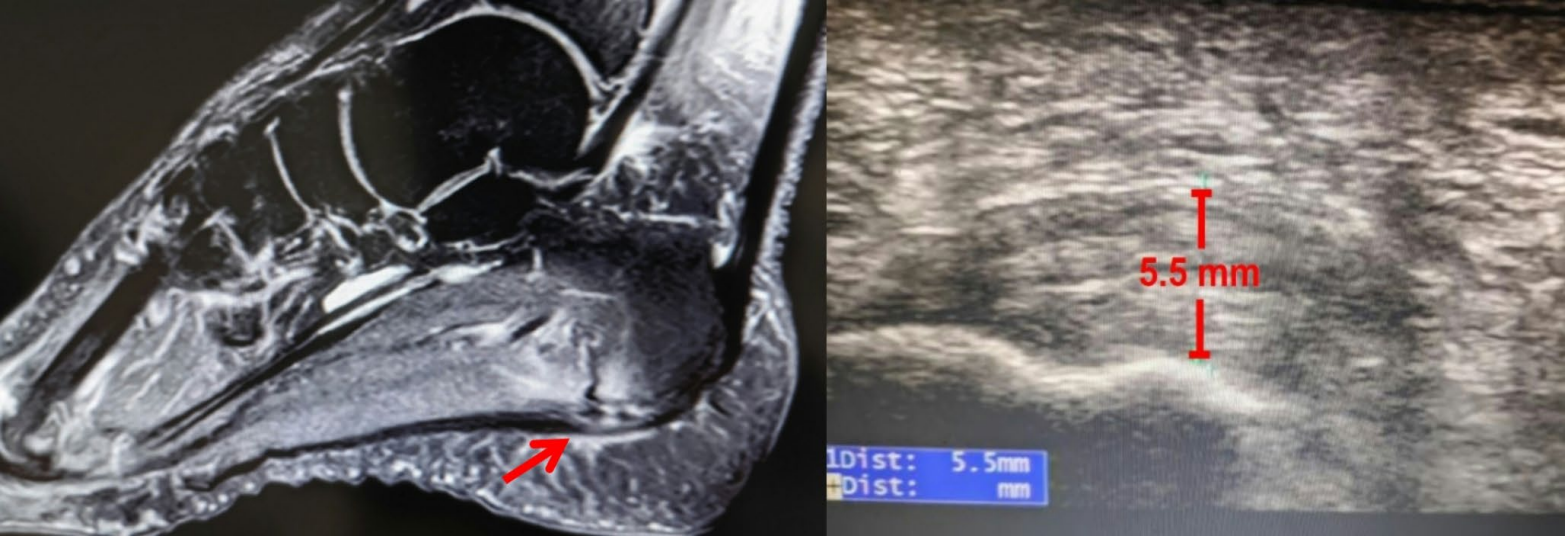
Preoperative MRI and ultrasound revealed an inflamed and thickened plantar fascia (red arrow). It indicative of chronic stress and inflammation. The MRI provided detailed visualization of soft tissue structures, confirming the extent of inflammation and any associated abnormalities. Ultrasound, as a dynamic and accessible tool, further highlighted the localized thickening and areas of increased vascularity, characteristic of plantar fasciitis

1. Patient with chronic refractory plantar fasciitis who does not respond to at least six months of standard conservative treatments, including biomechanical therapy (e.g., custom arch supports), extracorporeal shockwave therapy, stretching exercises, corticosteroid injections, or PRP injections.
2. Preoperative MRI confirming plantar fascia oedema or thickening, or ultrasound demonstrating plantar fascia thickening exceeding 4 mm or a greater than 20% increase relative to the contralateral side (Fig. 1).
3. First-time surgical intervention for refractory plantar fasciitis.
4. Age range of 30 to 60 years.
5. The follow-up period was at least one year.

#### Exclusion criteria

1. History of prior foot or plantar surgery.
2. Conditions such as Achilles tendinitis, calcaneal stress fractures, calcific tendinitis, Baxter’s nerve entrapment syndrome, foot neoplasms, or pathological fractures.
3. Congenital foot deformities such as flatfoot or foot arch instability.
4. Disease duration of less than six months or involvement in unavoidable heavy physical labor.
5. Local infection or cutaneous/subcutaneous inflammation.
6. Poor overall health status contraindicating surgical intervention.
7. Vascular diseases or a high risk of lower limb thrombosis.
8. BMI (Body Mass Index) exceeding 35.0 kg/m².

### Surgical method

#### Preoperative preparation

**Fig. 2:**
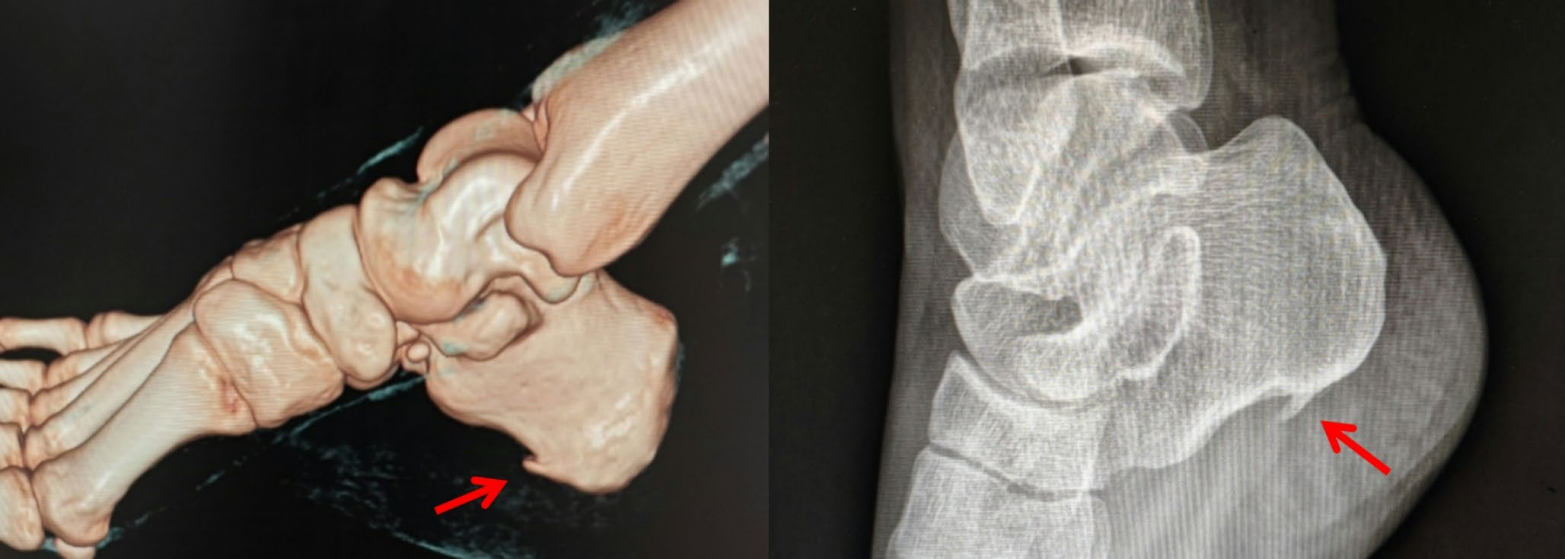
3D CT and X-ray images show calcaneal spurs (red arrow). Calcaneal spurs are bony outgrowths commonly associated with chronic stress or inflammation at the attachment sites of the plantar fascia or Achilles tendon

Prior to anaesthesia, tender points and surgical entry sites were meticulously marked on each patient’s foot. The dual medial deep fascia approach was employed for surgery, The posterior medial entry was located approximately 5 mm from the tangential line of the posterior edge of the medial malleolus to the plantar fascia (skin junction). The anterior medial entry was established two centimeters anterior to the posterior entry site. Preoperative X-rays and 3D CT scans can detect the presence of calcaneal spurs (Fig. 2).

#### Positioning and anesthesia

Patients were positioned supine with their affected leg arranged in a “4” position for optimal access during surgery. General anaesthesia was administered subsequently followed by routine sterilization protocols and draping of the lower limb; additionally, a tourniquet was applied to manage blood flow effectively.

#### Surgical steps

**Fig. 3:**
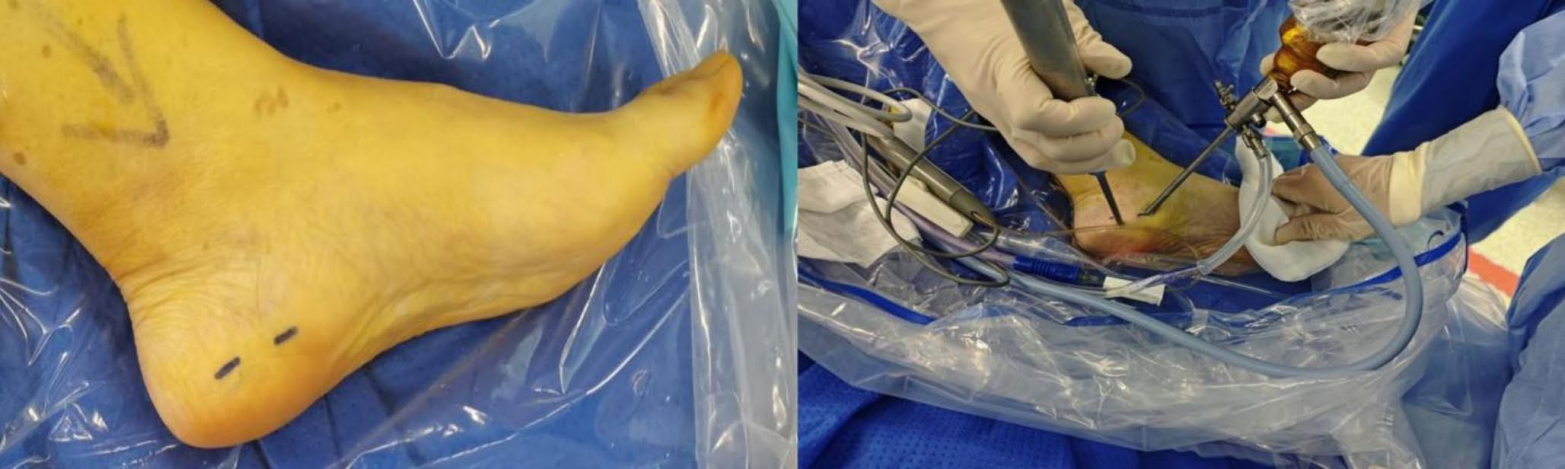
Preoperative incision marking and establishment of intraoperative approaches. Preoperative incision marking was performed to ensure accurate placement of surgical incisions, minimizing tissue damage and optimizing access to the affected area

**Fig. 4:**
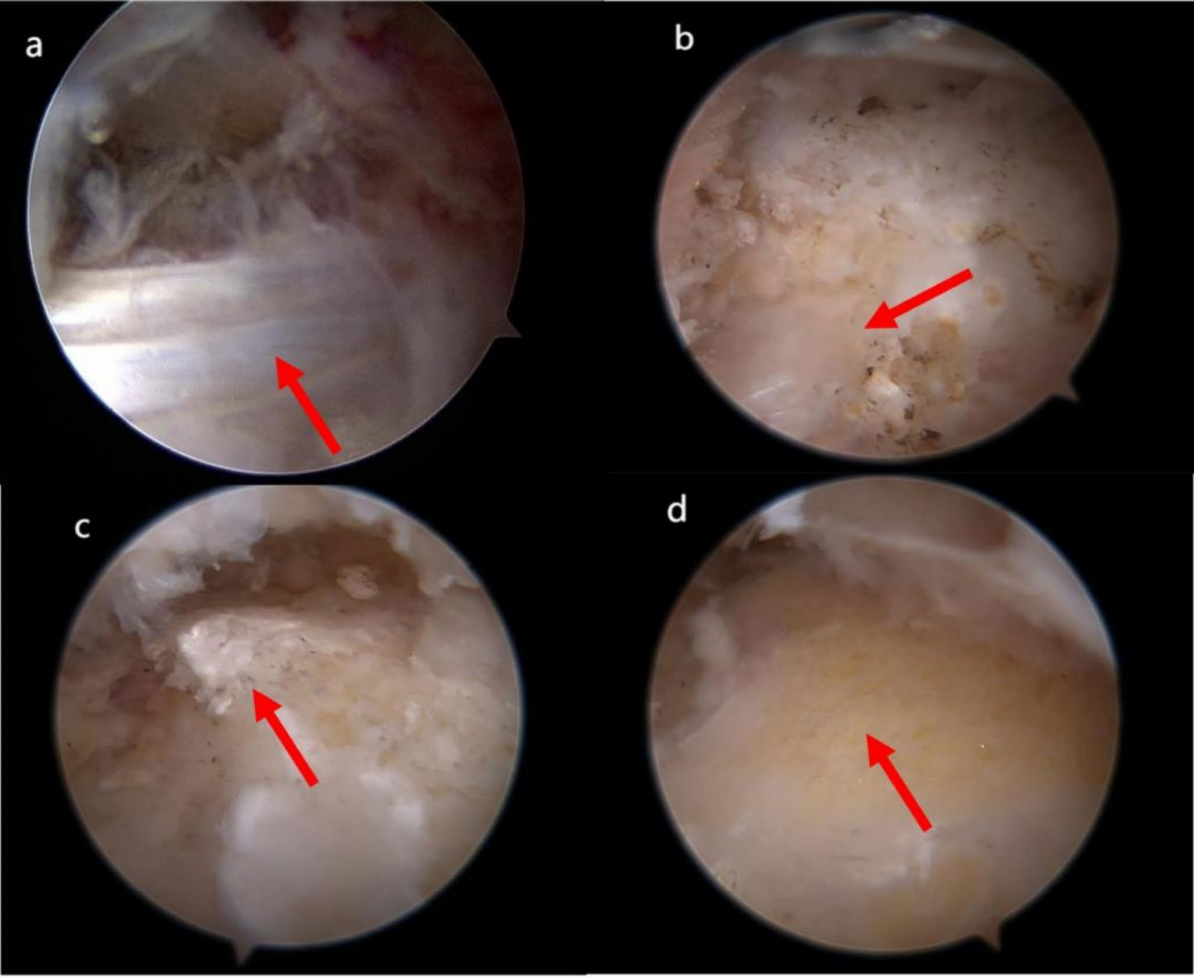
Intraoperative endoscopic procedures: (**a**) Inflamed and thickened plantar fascia; (**b**) Released plantar fascia; (**c**) Calcified bone spur on the bone surface; (**d**) Bone spur after removal (red arrow)

Tender points were accurately identified; a syringe needle facilitated precise localization of bone surfaces beneath soft tissue layers that required incision intervention—specifically creating an approximately 0.5 cm incision at each marked entry site for access purpose while blunt dissection using vascular forceps helps to adequately create the operative cavity and avoid nerve injury (Fig. 3). A four-millimeter diameter 30° endoscope was introduced through the posterior entry point, while plasma cutters along with shavers were introduced via the anterior entry. Excess tissue alongside any pooled blood were removed to improve visibility within operative fields, allowing identification of the calcaneal attachment of the plantar fascia and inflamed areas under endoscopic guidance (Fig. 4a).

The plantar fascia was released utilizing a shaver and plasma cutter, with the extent of release limited to one-third to one-half of the fascia, in accordance with standard recommendations. Surrounding inflamed tissue was excised, exposing subfascial fat tissue, which marked the completion of deep fascia release (Fig. 4b). Most patients presented with calcaneal spurs, and white calcified tissue was often observed at the attachment site of the plantar fascia (Fig. 4c). After preoperative fluoroscopy, a motorized burr was used intraoperatively to remove bone spurs (Fig. 4d), with caution taken to avoid excessive plantar fascia release during spur removal. Haemostasis was achieved through the use of the plasma cutter. Intraoperative fluoroscopy confirmed complete spur removal. For patients with significant calcaneal oedema shown on preoperative MRI, selective microfracture is performed with a 2 mm Kirschner wire. Subsequently, instruments were withdrawn, the wound was sutured closed, and a pressure dressing was applied prior to releasing the tourniquet.

#### Functional rehabilitation and postoperative considerations

**Fig. 5:**
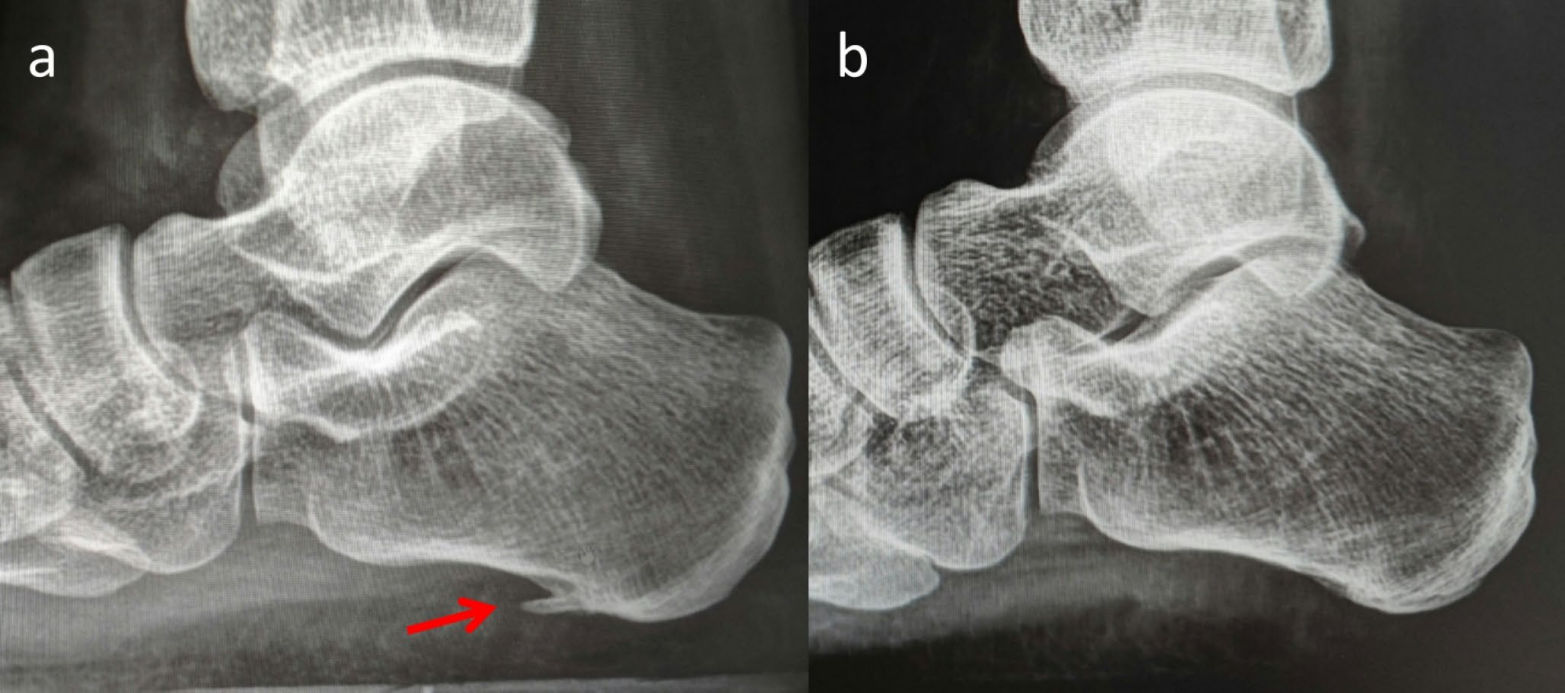
X-ray comparison before and after Calcaneal spur removal. (**a**) Preoperative X-ray shows a prominent bone spur beneath the calcaneus (indicated by the red arrow). (**b**) Postoperative X-ray shows that the bone spur has been removed, the plantar bony prominence has disappeared, and the bony structure appears smoother compared to the preoperative state

Postoperatively, routine dressing changes and anti-swelling treatments were administered for a duration of two weeks. Pre-discharge follow-up X-rays are used to observe the condition of bone spur removal (Fig. 5). Patients were encouraged to commence joint flexion-extension exercises on the first postoperative day and initiate weight-bearing exercises after one week. Although intradermal sutures are cosmetically advantageous, they have been associated with suture reactions in some patients that may delay recovery; therefore, standard sutures are recommended along with instructions for timely removal to facilitate rehabilitation. At the same time, advise patients with high BMI to undergo necessary weight management postoperatively.

### Evaluation

The primary indicators are the VAS, AOFAS score and the MLAA, while the secondary indicators include the NH/FL, AI, operation time, wound healing status, and number of complications. Basic information such as patient age, BMI, sex, presence of calcaneal spurs, smoking status, and rheumatoid diseases (especially ankylosing spondylitis) is also collected. The VAS and AOFAS scores were collected preoperatively and at one month, six months, and 12 months postoperatively.

**Fig. 6:**
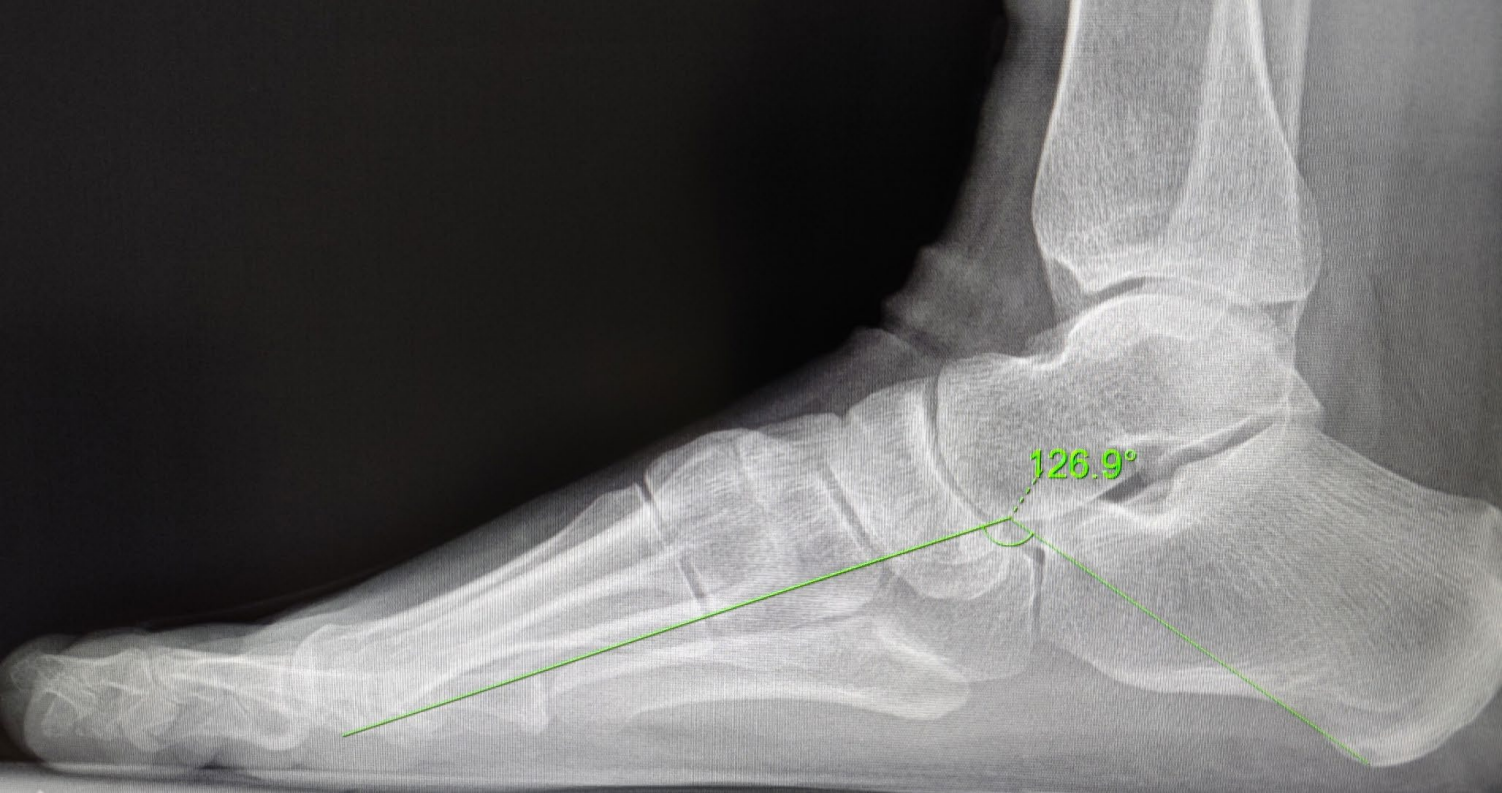
Measurement of the medial longitudinal arch angle. MLAA is the angle formed by the intersection of a straight line from the lowest point of the calcaneus to the lowest point of the talar head and another straight line from the lowest point of the talar head to the lowest point of the first metatarsal head

MLAA is the angle formed by the intersection of a straight line from the lowest point of the calcaneus to the lowest point of the talar head and another straight line from the lowest point of the talar head to the lowest point of the first metatarsal head on a weight-bearing lateral X-ray of the foot arch. Collected preoperatively and at 12 months postoperatively (Fig. 6).

NH/FL is the vertical height from the navicular tuberosity to the ground divided by the foot length, with the foot length defined as the distance from the heel to the longest toe. AI is similar to this, calculated as dorsum height/total foot length × 100, where dorsum height is the vertical distance from the superior edge of the navicular to the ground, and total foot length is the distance from the heel to the tip of the toes. Collected preoperatively and at 12 months postoperatively.

### Statistical methods

Functional outcomes were compared preoperatively and postoperatively, while structural outcomes were assessed between preoperative and final follow-up measurements. Additionally, differences in VAS scores, AOFAS scores, and changes in MLAA were analyzed between patients with calcaneal spurs and those without. Furthermore, a comparative analysis was conducted with relevant literature to assess operative time, pain and functional scores, the number of complication cases, and other related parameters.

Data were analyzed utilizing SPSS statistical software. Continuous variables were presented as means ± standard deviations (x ± s). The Shapiro-Wilk test was performed using software to assess the normality of the data. Paired t-tests were employed to compare preoperative and postoperative data. The Mann–Whitney U test was used to compare the mean age, AOFAS scores, and VAS scores at the final follow-up between the calcaneal spur group and the non-calcaneal spur group. Fisher’s exact test evaluated differences in gender and injury site distribution. Wilcoxon Signed-Rank tests were conducted to evaluate changes in MLAA from pre- to postoperative within each group. A *P*-value < 0.05 was considered statistically significant. Sample size calculations indicated that a minimum of 16 patients was necessary for this study.

## Results

**Fig. 7:**
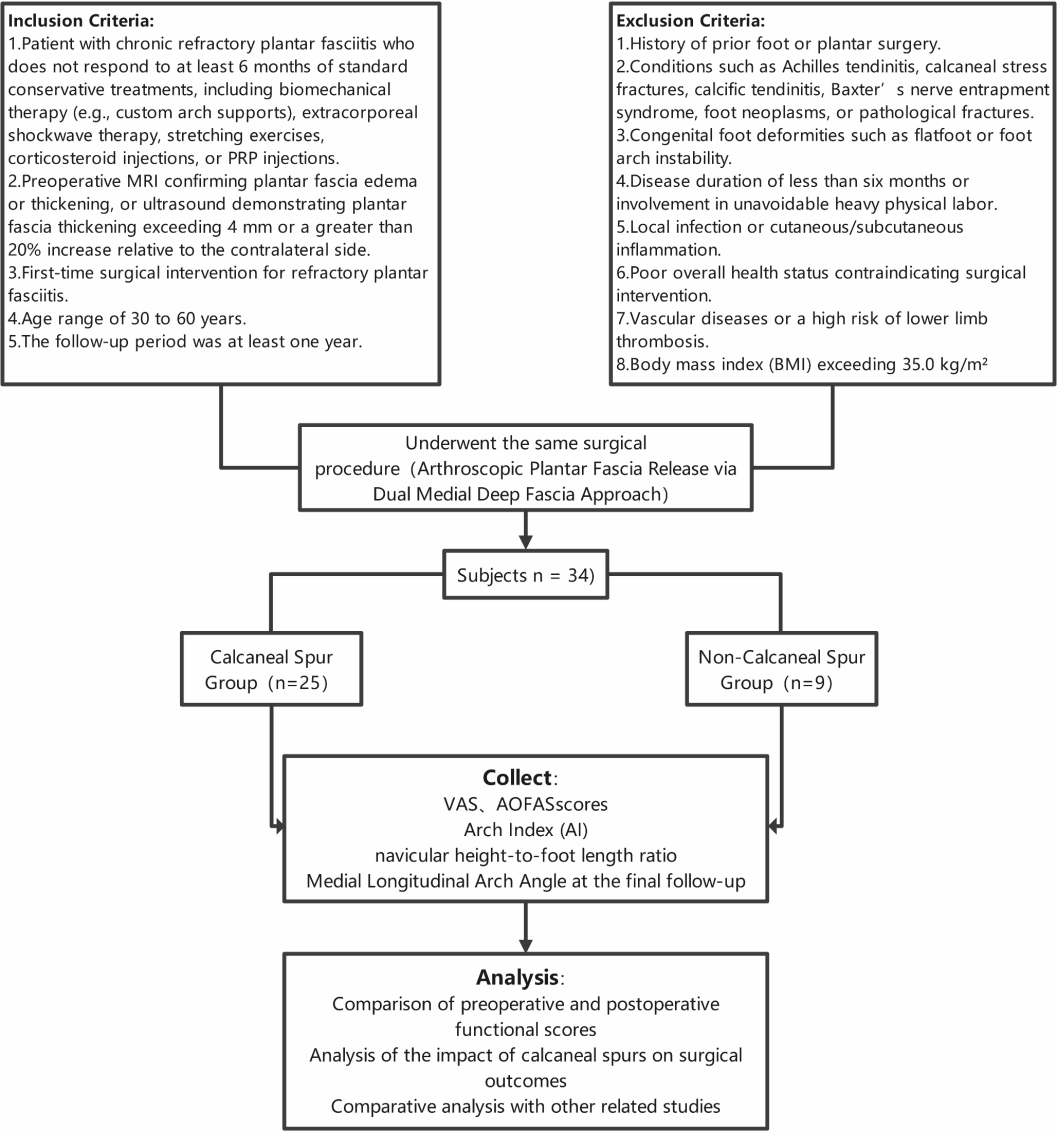
Study flowchart of patient selection and grouping. A total of 34 patients underwent endoscopic plantar fascia release, with relevant data collected and statistically analyzed

Through the above screening process and data collection analysis, we obtained many results (Fig. 7). A total of 34 patients were included in this retrospective study, consisting of 16 males and 18 females, with an age range of 31 to 55 years (mean age: 41.56 ± 6.64 years). The average BMI was 24.56 ± 2.16 kg/m²; notably, two patients (one male, one female) had a BMI exceeding 30 kg/m². Six patients had a smoking history, and one patient had a rheumatoid disease. All participants had a disease duration of more than six months. Preoperative imaging identified calcaneal spurs in 25 patients (Table 1).

**Table 1:** Patient characteristics

	Patient (*n* = 34)
Mean Age (years)	41.56 ± 6.64 (31–55)
Gender (male/female)	16/18
Calcaneal Spurs	25
Mean BMI (kg/m^2^)	24.56 ± 2.16
Smoking History	6
Rheumatoid Disease (Ankylosing Spondylitis)	1 (0)

**Fig. 8:**
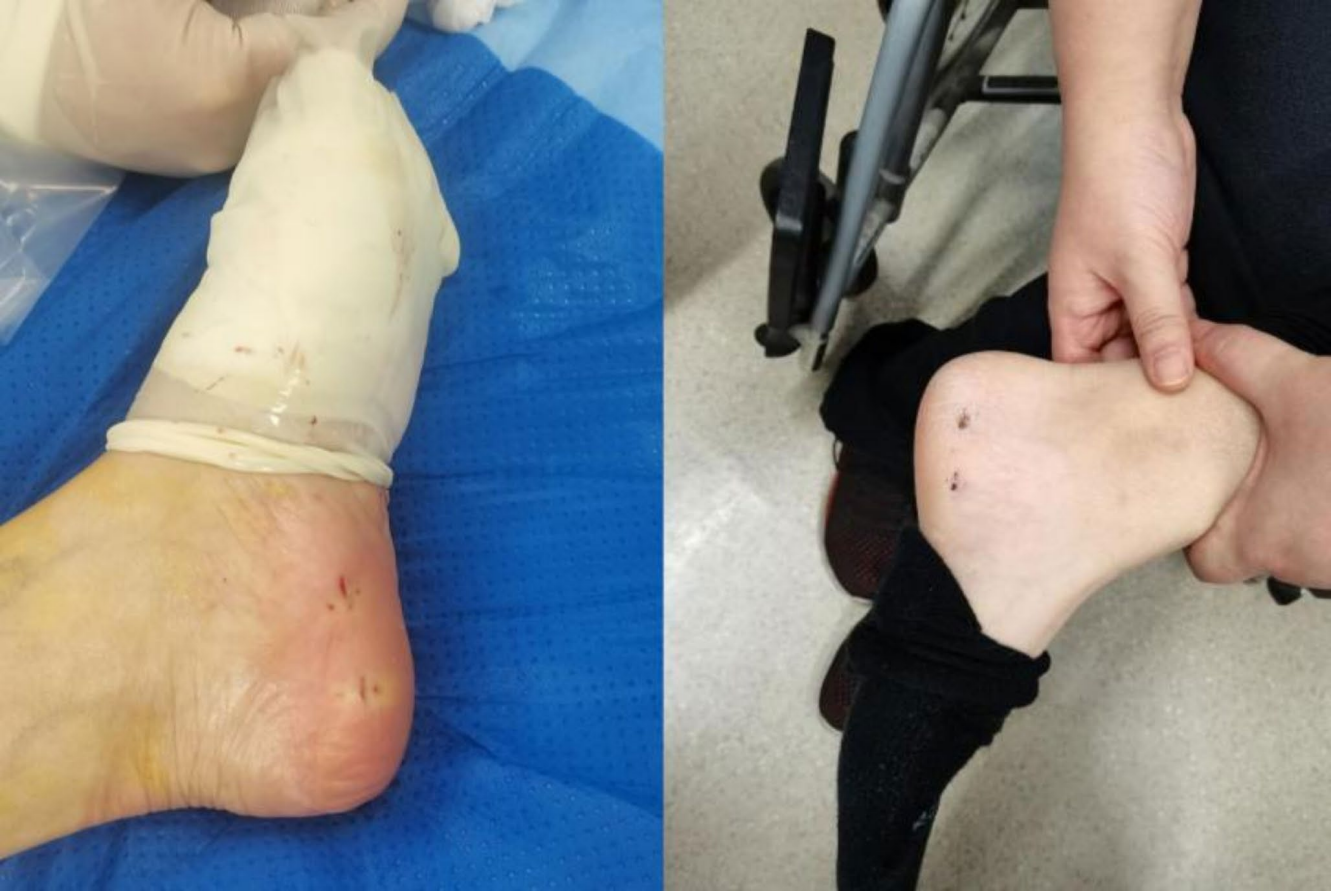
Postoperative and one-month postoperative skin incision. Postoperative evaluation of the skin incision showed proper closure with no signs of infection or dehiscence. At the one-month follow-up, the incision demonstrated further healing, with minimal scarring and no complications

The surgery was successful in all 34 patients, with an average operative time of 24.97 ± 4.37 (19–36) minutes. All wounds healed by primary intention (Fig. 8), with no instances of wound infections, suture reactions, vascular or tendon injuries, or other early complications. No long-term complications were observed, including arch collapse, recurrence of plantar pain beyond three months, or ankle joint stiffness. Two patients experienced lateral foot numbness and small toe abduction difficulty, which resolved within three months postoperatively.

All 34 patients completed the full follow-up, with no cases lost. Thirty patients attended in-person follow-up visits, while four were monitored via phone consultations and the final outpatient visit. During the follow-up period, two patients expressed dissatisfaction with their pain relief. At the final follow-up, all but one patient achieved satisfactory outcomes, with significant pain reduction or complete pain relief, good walking ability at both the ankle and heel, and normal physical function.

VAS and AOFAS scores demonstrated significant improvements compared to preoperative values (*P* < 0.05). At the final follow-up, VAS score decreased from an average of 6.53 ± 1.19 preoperatively to 1.18 ± 0.76 postoperatively, and AOFAS score increased from an average of 52.41 ± 5.23 to 93.29 ± 3.91, both showing statistically significant differences between preoperative and postoperative assessments (*P* < 0.05).

There were no significant changes noted in the MLAA (preoperative: 117.40 ± 2.83; postoperative: 117.38 ± 3.15), the NH/FL (preoperative: 0.2662 ± 0.0128; postoperative: 0.2660 ± 0.0117), and the AI (preoperative: 26.69 ± 1.29; postoperative: 26.61 ± 1.18), with *P*-values exceeding 0.05 for each measure respectively. The foot arches did not exhibit significant collapse, and most patients demonstrated normal functional recovery. Ultimately, all but one patient reported satisfaction with their surgical outcomes (Table 2). This particular patient, who did not present any comorbidities, had a relatively high body mass index (BMI) of 32.6 kg/m².

**Table 2:** Pre- and postoperative comparison of parameters

Timepoint	VAS Score	AOFAS Score	Medial Longitudinal Arch Angle (°)	Navicular Height / Foot Length	Arch Index
Preoperative	6.53 ± 1.19	52.41 ± 5.23	117.40 ± 2.83	0.2662 ± 0.0128	26.69 ± 1.29
1-Month Postoperative	3.56 ± 0.93	77.88 ± 8.10	-	-	-
6-Months Postoperative	1.88 ± 0.73	87.21 ± 5.14	-	-	-
12-Months Postoperative	1.18 ± 0.76	93.29 ± 3.91	117.38 ± 3.15	0.2660 ± 0.0117	26.61 ± 1.18
*P*-Value	< 0.05	< 0.05	> 0.05	> 0.05	> 0.05

**Note:***P*-values < 0.05 indicate statistically significant differences, while *P*-values > 0.05 indicate no significant differences. *P*-values compare preoperative results with those at 12 months postoperatively

There were no significant differences in gender distribution or affected side between the calcaneal spur and the non-calcaneal spur group. The average age of patients in the calcaneal spur group was significantly higher than that of those in the non-calcaneal spur group (*P* < 0.01). Additionally, there were no significant differences in the MLAA before and after surgery within each group. Furthermore, at the final follow-up, no significant differences were observed in VAS and AOFAS scores between the two groups (Table 3).

**Table 3:** Comparison between calcaneal spur and no calcanea spur groups

Variables	Calcaneal Spur Group)	No Calcaneal Spur Group	*P*-Value
Mean Age (years)	49.04 ± 4.41	34.56 ± 3.13	<0.001
Affected Side (Right/Left)	17/8	6/3	>0.05
Gender (Male/Female)	12/13	4/5	>0.05
Final Follow-Up VAS Score	1.16 ± 0.85	1.22 ± 0.44	>0.05
Final Follow-Up AOFAS Score	92.76 ± 4.25	94.78 ± 2.39	>0.05
Medial Longitudinal Arch Angle (°)			
Preoperative	117.60 ± 3.01	116.84 ± 2.31	
Postoperative	117.60 ± 3.26	116.78 ± 2.88	
*P*-Value	>0.05	>0.05	

**Note:***P*-values < 0.05 indicate statistically significant differences, while *P*-values > 0.05 indicate no significant differences

## Discussion

The findings of this study affirm that the dual medial deep fascial approach for endoscopic plantar fascia release yields excellent and safe therapeutic outcomes in patients suffering from refractory plantar fasciitis. Most participants exhibited significant functional improvements, characterized by substantial reductions in pain and enhanced mobility. Structural outcomes remained stable without significant changes; overall, ankle-foot function was restored to normal levels. Additionally, the operation time was shorter, and complications were fewer. The presence of calcaneal spurs does not impact postoperative outcomes.

In comparison to other surgical techniques, endoscopic minimally invasive treatment presents distinct advantages including smaller incisions, improved cosmetic results, and expedited recovery times [6]. Barrett and Day first introduced the application of endoscopy for treating plantar fasciitis in 1991 through a medial and lateral superficial fascia approach [13, 19]. Subsequent studies conducted by Bazaz, Ferkel, O’Malley et al. have corroborated the efficacy of this superficial fascia technique [20]. Subsequently, the study conducted by Komatsu et al. also confirmed the effectiveness of the deep fascial approach [9]. However, these earlier techniques were not without limitations, notably its lack of natural cavities can restrict operating space significantly. Additionally, patient positioning may hinder surgical visibility which complicates procedures and prolongs operation time [10]. Concerns regarding potential damage to the heel fat pad are also pertinent as such damage could exacerbate postoperative pain and delay recovery processes further still. Moreover, dual medial-lateral incisions might increase areas affected by postoperative pain complicating footwear use and impeding early rehabilitation efforts [21, 22].

Therefore, we compared the surgical outcomes with those reported in earlier studies. In terms of surgical time, our modified dual medial deep fascial approach required an average of 24.97 ± 4.37 (19–36) minutes, outperforming the surgical times reported for the deep fascial approach in previous studies. Regarding pain and function, the VAS and AOFAS scores at the final follow-up were significantly better than those from previous surgical methods indicating that our method more effectively reduced postoperative pain and achieved better recovery. In terms of safety, like previous surgical methods, there were no significant differences in structural scores before and after surgery, showing that we successfully maintained structural stability. Two cases of early complications were observed, with no long-term complications (Table 4). These outcomes are due to several key advantages of the dual medial deep fascial approach described in this study. First, most patients exhibited tenderness points on the medial side, with calcaneal spurs predominantly located medially at the calcaneal tuberosity [15, 23]. This anatomical consideration facilitates the surgical procedure and reduces operation time. Meanwhile, this approach provides a larger operative space and clearer surgical visualization. We use a syringe needle for drainage, tender point localization, and fascial release control, allowing for a clearer view of deep anatomical structures, more precise fascial excision, reduced risk of adjacent tissue injury, and lower risk of nerve damage, enabling more accurate surgical intervention. By minimizing tissue damage, this method also reduces pain and shortens surgical time. Furthermore, the dual medial incisions not only minimize postoperative pain, promoting faster recovery and facilitating rehabilitation, but also, along with the protective dressing, have minimal impact on footwear use, further aiding in postoperative mobility. Following rigorous patient selection and strict adherence to surgical techniques, we did not observe any cases of postoperative arch collapse. At the 12-month follow-up, almost all patients demonstrated normal functional recovery with significant improvements in clinical outcomes. However, there was still one patient with poor results, which may be related to their high BMI. Related research indicates that over 50% of patients with a BMI exceeding 27 kg/m² experience suboptimal results [18]. Therefore, postoperative weight management may also be an important factor influencing recovery in patients with high BMI.

**Table 4:** Comparative analysis of relevant literature

Study	Approach Type	Surgical Time (minutes)	Follow-up Duration	VAS Score (Pre-op → Final Follow-up)	AOFAS Score (Pre-op → Final Follow-up)	Sample Size	Complications
Our study	Dual Medial Deep Fascia Approach	24.97 ± 4.37 (19–36)	1 year	6.53 ± 1.19 → 1.18 ± 0.76	52.41 ± 5.23 → 93.29 ± 3.91	34	2 cases
Komatsu et al. (2011)	Dual Medial-Lateral Deep Fascial Approach	30–60	2 years	Not specified	64.2 ± 6.3 → 92.6 ± 7.1	10	3 cases
Miyamoto et al. (2017)	Dual Medial-Lateral Superficial Fascial Approach	30–50	4 years	Not specified	65.3 ± 5.0 → 91.1 ± 8.5	24	3 cases
Çatal et al. (2017)	Dual Medial-Lateral Deep Fascial Approach	35 ± 5.62	1 year	8.19 ± 1.03 → 1.81 ± 1.47	52.38 ± 10.23 → 82.71 ± 10.90	21	2 cases
Jerosch et al. (2004)	Dual Medial-Lateral Deep Fascial Approach	30–60	1 year	8.2 ± 1.1 → 2.1 ± 1.3	62.8 ± 5.6 → 88.3 ± 6.4	36	None
Blanco et al. (2001)	Dual Medial-Lateral Superficial Fascial Approach	50–60	1 year	Not specified	Not specified	38	5 cases

However, from an anatomical perspective, incorporating a medial approach may increase the risk of nerve injury. In this study, two patients experienced transient lateral plantar sensory numbness and small toe abduction difficulty, which may have been caused by lateral plantar nerve injury. Ogilvie-Harris and Lobo, Jerosch et al., and Miyamoto all pointed out that the medial approach may increase the risk of lateral plantar nerve damage. However, this risk can be mitigated through careful surgical technique. This includes limiting incisions to the skin and employing blunt dissection with vascular forceps to separate tissues up to the deep fascia layer. It is also crucial to minimize irritation to subcutaneous fat during surgery in order to reduce potential nerve damage risks. Further research into the precise anatomical course and variations of the lateral plantar nerve would be beneficial for optimizing incision sites.

The plantar fascia plays a crucial role in maintaining the structural integrity of the foot arch, and its biomechanical and pathological characteristics are vital for optimal ankle-foot function [24–26]. Therefore, it is essential to assess whether surgical release of the plantar fascia and removal of calcaneal spurs impact overall foot functionality. Most studies recommend that the release extent should not exceed 40%, and during the removal of bone spurs, excessive fascia release aiming for complete removal of the spurs should be avoided [27]. This minimizes the impact of surgical manipulation on the stability of the arch and maintains the normal biomechanics of the foot arch. In this study, the release extent for all surgeries ranged between a third and a half, and excessive plantar fascia release was avoided during bone spur removal, aligning with mainstream recommendations [28]. According to reports, the overall excellent and good rates of endoscopic treatment for plantar fasciitis range from 76 to 92%, with slight variations among different studies, but generally within this range [18, 29]. However, in our clinical practice, we have observed that the excellent and good rates of this modified technique may be even higher.

Both the calcaneal spur and non-spur groups demonstrated favorable surgical outcomes, with no statistically significant difference observed between the two groups. At the final follow-up, the average age of the calcaneal spur group was significantly higher than that of the non-calcaneal spur group, suggesting that calcaneal spurs are an age-related phenomenon, although further studies are needed to confirm this. Interestingly, while plantar fasciitis is often accompanied by calcaneal spurs [30], current research has not provided clear evidence directly linking these spurs to pain or thickening of the fascia. Some patients diagnosed with plantar fasciitis exhibit no visible spurs, whereas individuals without any symptoms may present with calcaneal spurs [16, 29, 31]. Studies have suggested that calcaneal spurs may develop as a compensatory mechanism [24]. Currently, most studies suggest that calcaneal spurs are generally unrelated to the symptoms or pathogenesis of plantar fasciitis [16, 24, 31, 32]. However, a small number of studies present opposing views, though their credibility remains low and requires further validation [17, 33]. Therefore, the relationship between calcaneal spurs and plantar fasciitis may warrant further investigation. Histopathological studies indicate that plantar fasciitis is primarily a degenerative condition resulting from repetitive microtrauma and subsequent healing processes, resembling tendinopathy rather than being solely an inflammatory response [25, 34, 35]. Nevertheless, some researchers suggest that the degenerative alterations in the plantar fascia may follow both acute and chronic inflammatory phases, where initial inflammation plays a role in facilitating later degeneration. Additionally, there is a fibrocartilaginous transition at the attachment site between the plantar fascia and the calcaneus, which helps distribute tension more effectively and evenly [24, 35]. Thus, understanding the pathogenesis and histopathology of plantar fasciitis remains a critical area for future research.

This study also has certain limitations, including a relatively small sample size and the retrospective nature of the study. Larger studies may uncover a wider range of patient outcomes. Furthermore, the absence of a control group restricts our ability to directly compare this technique with other approaches, although we performed a comparative analysis with relevant literature. Additionally, there may be technical limitations, such as an increased risk of lateral plantar nerve injury, which requires further research for verification. Nevertheless, the excellent results we obtained are undeniable.

## Conclusion

In conclusion, the dual medial deep fascia approach for endoscopic plantar fascia release is an effective, safe, reliable, and minimally invasive technique for treating refractory plantar fasciitis. It can reduce tissue damage, and allows for excellent functional recovery. The clinical outcomes of this approach are favorable, making it one of the best treatment options for refractory plantar fasciitis.

Furthermore, the presence or absence of calcaneal spurs does not significantly impact the postoperative clinical outcomes, indicating that this surgical method is effective regardless of the presence of spurs. The dual medial deep fascia approach offers several advantages, including reduced operative time and superior visualization compared to other techniques, leading to improved patient satisfaction and better recovery. The results of this study support the continued use and further exploration of this technique as one of the preferred treatment for refractory plantar fasciitis.

## Data Availability

No datasets were generated or analysed during the current study.
